# Benchmarking cEEGrid and Solid Gel-Based Electrodes to Classify Inattentional Deafness in a Flight Simulator

**DOI:** 10.3389/fnrgo.2021.802486

**Published:** 2022-01-06

**Authors:** Bertille Somon, Yasmina Giebeler, Ludovic Darmet, Frédéric Dehais

**Affiliations:** ^1^Artificial and Natural Intelligence Toulouse Institute, Université de Toulouse, Toulouse, France; ^2^Department for Aerospace Vehicles Design and Control, ISAE-SUPAERO, Université de Toulouse, Toulouse, France; ^3^Department of Psychology and Ergonomics, Technische Universität Berlin, Berlin, Germany; ^4^School of Biomedical Engineering, Science and Health Systems, Drexel University, Philadelphia, PA, United States

**Keywords:** electroencephalography, machine learning, Riemannian Geometry, flight simulator, inattentional deafness, Event-Related Spectral Perturbation (ERSP), mobile EEG, neuroergonomics

## Abstract

Transfer from experiments in the laboratory to real-life tasks is challenging due notably to the inability to reproduce the complexity of multitasking dynamic everyday life situations in a standardized lab condition and to the bulkiness and invasiveness of recording systems preventing participants from moving freely and disturbing the environment. In this study, we used a motion flight simulator to induce inattentional deafness to auditory alarms, a cognitive difficulty arising in complex environments. In addition, we assessed the possibility of two low-density EEG systems a solid gel-based electrode Enobio (Neuroelectrics, Barcelona, Spain) and a gel-based cEEGrid (TMSi, Oldenzaal, Netherlands) to record and classify brain activity associated with inattentional deafness (misses vs. hits to odd sounds) with a small pool of expert participants. In addition to inducing inattentional deafness (missing auditory alarms) at much higher rates than with usual lab tasks (34.7% compared to the usual 5%), we observed typical inattentional deafness-related activity in the time domain but also in the frequency and time-frequency domains with both systems. Finally, a classifier based on Riemannian Geometry principles allowed us to obtain more than 70% of single-trial classification accuracy for both mobile EEG, and up to 71.5% for the cEEGrid (TMSi, Oldenzaal, Netherlands). These results open promising avenues toward detecting cognitive failures in real-life situations, such as real flight.

## 1. Introduction

Neuroergonomics is a recent field of research that promotes the use of portable brain imaging to investigate complex cognitive processes that are difficult to observe and measure under laboratory settings (Parasuraman, 2003; Dehais et al., 2020a; Gramann et al., 2021). In this regard, recording systems have to fulfill certain requirements in terms of bulkiness, portability, sensitivity, and specificity (Hettinger et al., 2003; Somon et al., 2021). For instance, functional MRI (fMRI) or Magneto-Encephalography (MEG) strongly constrain volunteers freedom of movement to prevent signal contamination. They also often require long acquisition processes and the presentation of basic stimuli in a repetitive fashion due to the low signal-to-noise ratio (SNR). These settings negatively affect participants' motivation and render difficult the reproduction of critical real-life phenomena. One such phenomenon is inattentional deafness: the propensity to remain unaware of unexpected but perfectly audible sounds (Macdonald and Lavie, 2011; Dalton and Fraenkel, 2012). The occurrence of these failures of auditory attention has been shown to have devastating consequences in real life scenarios such as in the aviation or medical domains (refer to Dehais et al., 2019c for a review). In experimental contexts, inattentional deafness has been assessed through several experimental paradigms including auditory oddball paradigms, during which the participant is required to detect infrequent target sounds and ignore frequent distracting ones. At the behavioral level, inattentional deafness is characterized by an increased miss rate (undetected target sounds) compared to hits (detected targets). At the neurophysiological level, using this kind of paradigm, Molloy et al. (2015) used MEG to investigate the underlying neural mechanisms supporting inattentional deafness. Despite interesting findings, they failed to induce auditory misses in a lab context, preventing them from performing the expected contrast (i.e., hit vs. miss). To the authors' knowledge, only one lab study managed to identify the neural correlates of inattentional deafness with fMRI (Durantin et al., 2017). To this aim, the authors placed their participants in a challenging aerobatic flight scenario using goggles and a joystick placed outside of the fMRI. They obtained a 35% auditory miss rate yielding them to discriminate evidences of the activation of an attentional bottleneck mechanism that, in return, inhibits the auditory cortex when sounds failed to reach an awareness.

In the frame of neuroergonomics, electroencephalography (EEG) represents an alternative approach to observing this phenomenon under more ecological settings. Following this approach, Dehais et al. (2019c) equipped their participants with a research grade EEG system in a realistic motion flight simulator and obtained a 58% miss rate using a modified auditory oddball paradigm. At the event-related potentials (ERP) level, their results disclosed usual oddball-related activities: *i)* a negative N1 component, peaking frontocentrally 100 ms after both frequent and target sounds display, which is associated with the physical and temporal characteristics of the stimuli (Näätänen and Picton, 1987), and *ii)* a positive P3 ERP, emerging at parietal sites roughly 350 ms after target sound display, which is associated to the cognitive and higher order processing of the stimulus (Segalowitz and Barnes, 1993; Luck and Kappenman, 2011; Justen and Herbert, 2018). Interestingly, they also observed a lower N1 and P3 amplitude for auditory misses compared to hits. The high miss rate allowed them to implement machine learning techniques to classify hits vs. misses with 70% of accuracy, paving the way for the implementation of neuroadaptive technology in the cockpit (Fairclough and Lotte, 2020). However, Dehais et al. (2019c) used a cumbersome EEG system that might not be suitable for everyday life operations since it requires *i)* very long and sometimes painful set-up time, and *ii)* the use of a conductive gel which can be inconvenient and may dry over time, thus, lowering the SNR (Di Flumeri et al., 2019). Recent technological advances have allowed the development of gel-free pre-amplified dry-electrode systems providing freedom of movement for the users and even enabling the on-line streaming and processing of electrophysiological data (Di Flumeri et al., 2019). Such systems were used in real-flight conditions to investigate inattentional deafness to auditory alarms and provided both consistent and complementary findings to better understand the onset of this phenomenon (Callan et al., 2018; Dehais et al., 2019b). Despite their success to measure the brain in the wild, dry electrodes remain uncomfortable and even painful when worn over long periods of time (usually more than 40 min) as suggested by the participants of these studies, and observed on a comfort evaluation in Di Flumeri et al. (2019).

Fortunately, other solutions have arisen to monitor brain activity in the most transparent way for the user. For instance, the cEEGrid (TMSi, Oldenzaal, Netherlands) system is a ten-printed-electrode flexible fixed around the ear EEG. Similar to usual cap-EEG, they have been used to investigate EEG activity in lab conditions (Bleichner et al., 2016), during sleep (Sterr et al., 2018), walking (Hölle et al., 2021) but also driving (Wascher et al., 2019). Yet, their portability and quick set-up time make them more suitable for mobile EEG measures (Di Flumeri et al., 2019; Somon et al., 2021). In addition to long-term frequency measures (Sterr et al., 2018; Wascher et al., 2019), the cEEGrid has also proven efficient to record usual ERPs (Debener et al., 2015) showing similar components to usual cap EEG associated with oddball paradigms and visual Simon task (Pacharra et al., 2017). Even though the cEEGrid only bears 16–20 electrodes in the processing stage [depending on the amplifier used; Debener et al. (2015); Sterr et al. (2018); Somon et al. (2019)], several usual data-processing algorithms (i.e., ASR or ICA) can still be used for artifact removal (Bleichner and Debener, 2019; Blum et al., 2019). This gel-based unobtrusive system seems very fit for neuroergonomics studies over many hours with steady impedances (Debener et al., 2015). Yet it has to be mentioned that, unlike usual cap-EEG, the cEEGrid has very specific electrode locations, rendering the use of certain signal processing tools difficult, or the study of certain activities (i.e., from the prefrontal cortex) impossible. Finally, the data course recorded at these electrode sites can be different from those recorded at standard 10–20 electrode sites, even though they have been correlated with cap-EEG recorded ERP (Bleichner et al., 2016; Pacharra et al., 2017). Indeed, the cEEGrid (TMSi, Oldenzaal, Netherlands) also showed ERP patterns different from usual ones when considering the various electrode locations (e.g., bottom electrodes in Debener et al., 2015).

Alternatively, solid gel electrodes may provide a good compromise in terms of SNR, user comfort, and ease of use (von Lühmann et al., 2017; Di Flumeri et al., 2019). They have been used in few studies and show, similarly to the cEEGrid, a high degree of usability, comfort (Di Flumeri et al., 2019), and resistance measures over several hours [lowest impedance 4 h post-setup compared to dry and pasted electrodes; Toyama et al. (2012)]. Unlike the cEEGrid, dry electrodes are usually located on cap-EEG, thus, removing the difficulty of electrodes locations, thereby ERP time-course, differences. In addition, Di Flumeri et al. (2019) observed consistent impedance, but also power spectral density measures across time. Solid-gel based electrodes, thus, seem very fit for real-life scenarios recording. Two difficulties concerning the use of solid-gel, compared to cEEGrid EEG, still remain: *i)* data presented in these studies only portray spectral activity, not revealing the efficiency of solid-gel to measure and analyze temporal (ERP) and time-frequency (ERSP) data (Di Flumeri et al., 2019); and *ii)* unlike the cEEGrid, their obtrusiveness leads to decreased transparency still disrupting the engagement of participants in more ecological tasks.

In the present study, we test the feasibility to measure the electrophysiological correlates of inattentional deafness in a flight simulator using two different comfortable unobtrusive EEG systems. We performed concurrent EEG recording with a cEEGrid system placed around each ear and solid gel electrodes spread over the scalp. The experimental scenario consists in performing two critical approaches and landings similarly to Dehais et al. (2019c). Along with the flying task, pilots are presented with an auditory oddball and have to click on the side-stick trigger when they hear odd/deviant sounds. Our first objective is to determine whether we can extract time, frequency and time-frequency domain features over the EEG signals from the cEEGrid and the solid gel electrodes to discriminate auditory misses and hits at the statistical level. More precisely, according to the literature, we expect to detect both the oddball-related N1 and P3 ERP components with the solid-gel-based electrodes and the cEEGrid system (Hölle et al., 2021). We also expect variations at the frequency and time-frequency levels taking the form of increases in the alpha (α)—repeatedly associated with hypovigilance and fatigue (Campagne et al., 2004; Borghini et al., 2014)—and theta (θ) activity with increasing fatigue and time on task (Craig et al., 2012). These patterns, already observed in real flight and flight simulation studies (Poussot-Vassal et al., 2017; Dehais et al., 2019a) with usual cap EEG, associated with the consistency of spectral variations observations across time for both the cEEGrid (Sterr et al., 2018) and solid-gels (Hölle et al., 2021) seem relevant candidates for oddball responses discrimination.

Going further in the frame of neuroergonomics, a second objective is to test the feasibility of an off-line passive Brain-Computer Interface (pBCI) to infer inattentional deafness and to compare the accuracy of the two EEG systems. The experimental conditions, involving a motion flight simulator, represent a relevant test-bed to assess the signal quality of the two EEG systems given that the flying task and the flight simulator environment are respectively prone to muscular artifacts (eyes, head, and arms movements) and electromagnetic contamination.

## 2. Materials and Methods

### 2.1. Participants

Eleven participants took part in this experiment (2 women, 10 right-handers, age 23±2.05 y.o.). They were healthy, had no visual or hearing impairment as attested by their flying certificate, and were not under any medication. All the participants were familiar with piloting: they were either undergoing or had passed the French piloting license (PPL). The study was approved by the Institutional Review Board of the local French ethics committee (Comit d'Evaluation Ethique de l'Inserm, IRB00003888-18-460) and conducted according to the principles expressed in the Declaration of Helsinki. Participants provided a written informed consent prior to the experiment.

One participant was removed from further analyses due to difficulties using the flight simulator and performing the required tasks.

### 2.2. Experimental Tasks and Procedure

During this experiment, participants had to perform a flight simulation composed of two runs with varying visibility levels. During both runs, they were asked to perform a usual auditory oddball task, concurrent to the piloting task.

#### 2.2.1. Oddball Task

During the experimental session, participants were presented with an auditory oddball task. They had to ignore the frequent non-target sounds and use the trigger of the side-stick to respond to the auditory targets. The frequent and odd sounds were two different auditory stimuli (chirp sounds - *F*_*S*_ = 44, 100 Hz, 0.1 s duration) presented to the participants: an increasing-frequency sound (up-chirp) and a decreasing one (down-chirp) (Artieda et al., 2004). Sounds were generated with MATLAB (The MathWorks, Natik, USA) and displayed using the Psychophysics Toolbox extensions (Brainard, 1997; Kleiner et al., 2007). The inter-stimulus interval was set at 1.5 s to which a random jitter drawn from the standard uniform distribution between 0 and 2 s was added. Standard sounds were presented with a 75% rate and odd sounds with a 25% rate. The type of sound (i.e., up-chirp and down-chirp) associated with standard and odd sounds was counterbalanced across participants (5 participants had the up-chirp as the target, and 5 had the down-chirp), but stayed the same across the two runs (with the two visibility) for each participant. The total number of sounds presented varied across participants according to the time it took them to perform the simulation. The average ± SEM number of sounds across participants is presented in Table 1.

**Table 1:** The average number of sounds (± SEM) presented to the participants during flight simulation for each run.

	**Odd**	**Standard**	**Total**
Flight simulator	141.3 ± 5.13	423.4 ± 14.48	564.7 ± 18.9
Normal visibility	68.4 ± 3.74	210.1 ± 9.51	278.5 ± 12.72
Low visibility	72.9 ± 1.96	213.3 ± 5.88	286.2 ± 7.53

#### 2.2.2. Flying Simulator and Scenarios

##### 2.2.2.1. Flying Simulator

**Figure 1:**
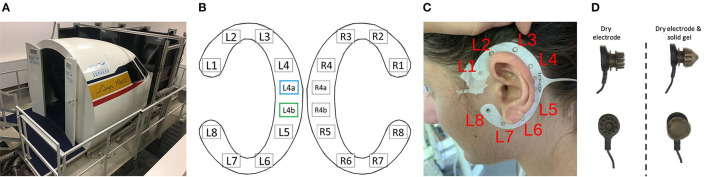
**(A)** Three-axis motion flight simulator at ISAE-SUPAERO. **(B)** Localization of the cEEGrid electrodes for both ears with recording reference (blue) and DRL (green) electrodes indicated. Electrodes R4a and R4b on the right grid were not recorded on our set-up. Layout adapted from the cEEGrid plugin (Martin G. Bleichner, 2019) in EEGLAB (v.2019.1) (Delorme and Makeig, 2004). **(C)** Localization of the left ear grid when fitted around the ear of a participant after cleaning and preparing the skin of the participant. **(D)** Illustration of a dry electrode with pins from the Enobio device (left) and the same electrode encapsulated with solid gel that has the consistency of silicone (right) to avoid discomfort or even pain.

The flying task was performed using the ISAE-SUPAERO three-axis (roll, pitch, and height) motion flight simulator designed by the French Flight Test Center which simulates a twin-engine aircraft flight model. It is composed of the classical actuators (side-stick, rudder, throttle, flaps lever, and autopilot), and displays the simulated environment on eight screens disposed of in a semi-circle outside the cockpit (refer to Figure 1A). Inside the cockpit, the user interface is composed of a Primary Flight Display, a Navigation Display, and an Electronic Central Aircraft Monitoring Display page. Two stereophonic speakers located under the displays on each side of the cabin are used to broadcast continuous engine sounds and to trigger the oddball sounds. Finally, the flight simulator environment enables the configuration of the visual environment (e.g., the moment of the day, visibility, wind and weather conditions).

##### 2.2.2.2. Flying Scenario

Participants had to perform two flight scenarios (i.e., runs) which consisted in performing an approach under two different visibility conditions (i.e., low vs. normal visibility). Both scenarios were composed of an approach and landing on the right runway of Toulouse-Blagnac airport (LFBO-14R). This scenario is an adaptation of the VOR-DME arc approach and landing, and has already been tested in a previous experiment successfully inducing inattentional deafness (Dehais et al., 2019c). The aircraft position was initialized at 20 nautical miles (NM) from the airport, at an altitude of 4, 000 feet (ft), a heading of 345°, and a speed of 170 knots (kts). First, participants had to make a U-turn on the left to come back to a 200° heading while descending to an altitude of 3, 000 ft. When reaching a 12 NM distance from the airport, participants had to turn to the west (heading 270°) until they intercepted the runway axis using the instrument landing system (ILS–a system that sends radio waves to guide the pilot to the runway). Once intercepted, the pilots had to take a heading of 144° in order to line up with the center line of the runway. At a 5 NM distance from the landing ground, they had to reduce speed to 130 kts to initiate the final descent until they landed on the runway. To increase the workload in both scenarios, we added a crosswind component throughout the flight, which made the task of heading tracking more demanding.

Finally, we manipulated the visibility in order to introduce variations in the runs and to avoid training effects. The repetition of these approaches and landings also allowed us to maximize the number of episodes of inattentional deafness (these final flight phases being particularly demanding and engaging Dehais et al., 2019c), which is of great importance to improve the SNR for the electrophysiological analyses (i.e., ERPs). The two visibility levels were introduced as separate runs one after the other in random order. In the normal visibility (NV) run, there was a clear sky with no clouds. In the low visibility (LV) run, a very thick layer of clouds was implemented to decrease the visibility on the landing ground. This manipulation is not expected to induce an increased cognitive load, as participants were performing instruments-based landings which require focusing on flight parameters until the runway appears at the very last moment of the scenario in the two cases. Each run took roughly 15 min (14.57±0.34 min on average) to perform.

#### 2.2.3. Procedure

Upon their arrival, participants were asked to read and sign the informed consent form stating that they were willing to take part in the experiment. After a quick overview of the imminent tasks, the two cEEGrid (TMSi, Oldenzaal, Netherlands) for the right and left ear (refer to Figures 1B,C) were positioned according to the manufacturer and literature recommendations (Mirkovic et al., 2016; cEEGrid - Stefan Debener, 2019). After verifying the impedance of the electrodes and data quality on the BrainVision Recorder software (Brain Products GmbH, Gilching, Germany), the 8-dry-electrode Enobio cap was positioned and electrode locations (Fpz, Fp1, Fp2, Fz, Cz, Pz, C3, and C4) were verified to fit the International 10–20 layout (Jasper, 1958). Next, impedance and data quality were checked on the NIC2.0 (Neuroelectrics, Barcelona, Spain–v2.0.11.1) software. Participants were finally installed in the flight simulator where they were provided the exact flying and approach procedure (i.e., the VOR-DME arc approach described in paragraph 2.2.2.2).

Participants were not familiar with the VOR-DME arc approach, and had never performed it before. Consequently, they were asked to perform a first familiarization phase—without motion—during which they got used to the instruments (i.e., the PFD and ND, the ILS and VOR systems) and dynamics of the flight simulator. This phase was shorter than the experimental flying scenarios, as the aircraft's starting position was at the 200° heading, 15 NM from the airport. No wind was added during the familiarization phase, and the visibility was clear. During this phase, participants were also trained with the oddball sounds and the side-stick trigger they had to press every time they heard a target/odd sound.

When participants felt comfortable with the flight simulator, the experimental phase was launched. The order of the two runs was counterbalanced across participants: 5 started with the low visibility scenario and 5 with the normal visibility one. An emphasis was made for all participants on the fact that no task (flight simulation or oddball) should be prioritized over the other.

## 3. Measures and Analyses

All the behavioral and physiological data were streamed, recorded, and synchronized through the LabStreaming Layer and associated apps (Kothe et al., 2019).

### 3.1. Behavioral Data

Participants' behavioral responses were continuously streamed from the flight simulator *via* LSL at a 100 Hz frequency rate. Response type and reaction times (RT) were extracted and analyzed *a posteriori* with MATLAB (The MathWorks, Natik, USA). There were on average (mean ± SEM) 75.5±0.65% standard and 24.5±0.65% odd sounds for the normal visibility task, and 74.5±0.32% standard and 25.5±0.32% odd sounds for the low visibility scenario. On average, less than 3 false positives (i.e., responding to a standard sound) and late responses (i.e., responding to an odd sound more than 2 s after sound display) were observed per participant. Based on these, hit and miss rates were computed for each participant, in every condition (i.e., low and normal visibility), respectively, as the number of correctly detected targets and the number of targets not responded to over the total number of odd sounds. In addition, based on the Signal Detection Theory, the *d'* coefficient was computed as a measure of sensitivity to the sounds for each experimental condition (Swets et al., 1961; Anderson, 2015). Finally, RT were computed as the time delay between the sound display and the participant's response. Late responses (*RT*>2 s) were not considered in the analysis.

Miss rate, *d'* and RT were analyzed with MATLAB (The MathWorks, Natik, USA) with a pairwise *t*-test with visibility (low visibility vs. normal visibility) as a within-subject factor. In addition, for each condition, the difference of the *d'* from 0 was tested with a *t*-test, showing whether odd sound detection was higher than chance or not. All results are reported as mean ± SEM.

### 3.2. Electroencephalography

In order to assess the usability and comfort of several mobile EEG recording systems, two different systems were tested: a cEEGrid (TMSi, Oldenzaal, Netherlands) system and a dry-electrode Enobio (Neuroelectrics, Barcelona, Spain) system. The electroencephalography (EEG) was continuously recorded and synchronized with the LabStreaming Layer from these two systems.

#### 3.2.1. cEEGrid EEG Recording System

cEEGrids are C-shaped around the ear adhesive 10 Ag/AgCl arrays which allow recording electrical brain activity non-invasively, unobtrusively, and are very portable (Bleichner et al., 2016). On each grid (left and right ear), one electrode can be used as reference (L4a/R4a respectively on the left and right ear) and one other as driven right leg, or DRL (L4b/R4b–refer to Figure 1B). The other 8 electrodes on each grid (L1-L8 and R1-R8) are recording channels. On our set-up (refer to Figures 1B,C), the L4a and L4b electrodes were the references and DRL electrodes for both grids.

After cleaning and preparing the skin around each ear with an abrasive paste and alcohol, a small amount of electrolyte gel was applied on each of the 10 electrodes of each grid (as recommended on the cEEGrid website cEEGrid - Stefan Debener, 2019). The two grids were then positioned around each ear with a double-sided adhesive and hard-wire-connected together and onto an adaptor (ActiCap) specifically designed to connect the cEEGrids to a LiveAmp Bluetooth 24-bit DC-amplifier (Brain Products GmbH, Gilching, Germany). As mentioned in Mirkovic et al. (2016), electrode locations can vary slightly from one participant to another. Data were collected wirelessly *via* Bluetooth at 500 Hz through the LSL LiveAmp Recorder app.

##### 3.2.1.1. Pre-processing

cEEGrid data were analyzed using EEGLAB software (v.2019.1) (Delorme and Makeig, 2004) on MATLAB (The MathWorks, Natik, USA)(v.2019a). Several steps were performed to *i)* extract spectral bands of interest, *ii)* denoise and clean data and, *iii)* epoch data.

First, raw EEG data were down-sampled to 250 Hz and band-pass filtered (FIR using Hamming window with order 414 and 1 Hz transition bandwidth). Cutoff frequencies were adapted according to the requirements for further analyses: [1−20] Hz for ERP analyses and [1−40] Hz for frequency and time-frequency analyses. Due to the ecological context and the nature of the cEEGrid electrodes, an extensive and carefully designed denoising procedure has to be performed. Artifact Subspace Reconstruction (ASR) was used as a primary step as it has been shown to be very effective in cleaning data from large amplitude artifacts (Chang et al., 2018, 2019; Miyakoshi and Kothe, 2019). ASR procedure removes artifacts based on a Principal Component Analysis (PCA) of the covariance matrices of the data on sliding windows. Covariance matrices that exceed the pre-defined threshold are projected (e.g., interpolated) on the leading components of the PCA. We used ASR implementation from the *clean_rawdata* function of the *clean_rawdata* plugin (ver.2.3) with the following hyper-parameters: *highpass* set to off, *flatline* to 5 s, *channel correlation* to 0.85, *line noise* to 4 SDs, *burst* to 10 SDs, and *maximum repaired time windows* to 0.45 (i.e. 45%). Hyper-parameters were set following defaults recommended in the *clean_rawdata* function. We only increased the burst and window criteria due to noisier data in the flight simulator and increased motion artifacts. Next, missing channels were interpolated and, following the procedure in Hölle et al. (2021), data were re-referenced to the 6 and 7*th* electrode of each grid (L1 to L4 were re-reference to the average of L6 and L7, and R1 to R4 were re-referenced to the average of R6 and R7). This procedure allows to analyze vertical bipolar derivations, and is expected to yield the highest amplitude (Debener et al., 2015; Bleichner et al., 2016). Next, data were corrected for remaining artifacts through a standard Independent Component Analysis [ICA; Makeig et al. (2004)]. Components corresponding to artifacts were automatically detected and removed using the ICLabel plugin (Pion-Tonachini et al., 2019): components labeled at more than 70% as eye, muscle, bad electrode, or other artifact components.

Finally, data were epoched time-locked to the sound stimuli, between −200 and 1, 000 ms around the standard and odd sounds for ERP analyses, and between −1 and 1.5 s around the standard and odd sounds for spectral analyses. Epoched data for ERPs were baseline-corrected in the −200 to 0 ms time-window before the stimulus.

Two participants had to be removed from the cEEGrid analysis due to large noisy portions of the signal, therefore unusable, after the pre-processing steps described above. For the remaining 8 participants, epoched data were averaged for each experimental condition separately according to the type of stimulus and response of the participant to target sounds (hits and misses) but also to the visibility level (normal vs. low visibility). Usual ERPs and power spectral densities (PSD) were computed. PSD was obtained with a Welch periodogram using a 250 ms window and no overlap. Finally, time-frequency measures (Event-Related Spectral Perturbations–ERSPs) were computed with wavelets implementation from EEGLAB (v.2019.1) (Delorme and Makeig, 2004) with 40 frequencies in the [3−40] Hz window, 3 wavelet cycles at the lowest frequency, an increasing factor of .8 and 250 time-points in the −442 to 938 ms time window. At first, we observed the data according to the stimulus-response pattern to target sounds (hits vs. misses) and visibility (normal vs. low visibility) to gain insights into inattentional deafness, related activity. In addition, to avoid differences related to the number of trials for spectral activity data, an equal number of trials was randomly selected in each condition at the participant level for frequency and time-frequency analyses only. In the end, there were on average 42.63±5.14 trials per participant. Thus, the final analysis focuses on statistical comparisons according to the stimulus-response pattern to odd sounds.

Finally, according to previous cEEGrid studies on oddball paradigms, data at the L2-L3 and R2-R3 electrodes were averaged and analyzed separately for left and right grids (Debener et al., 2015; Hölle et al., 2021). In accordance with the oddball-related and previous cEEGrid studies (Debener et al., 2015; Somon et al., 2019), visual inspection of ERP data revealed a negative component around 150 ms—the N1 ERP, having a later peak on cEEGrid data compared to usual EEG caps—and a broader positive one starting around 300 ms after sound display—the P300 component. The P300 is computed as the average amplitude in the [300−500] ms post-stimulus time window. ERPs, PSDs, and ERSPs were compared for the odd sounds (hits vs. misses) at each time and/or frequency point with EEGLAB (Delorme and Makeig, 2004) through a permutation test with False Discovery Rate (FDR) correction for multiple comparisons. The statistical significance was set at α = 0.05.

#### 3.2.2. Enobio EEG Recording System

An 8-dry-electrode Enobio wireless recording system was used in this experiment in addition to the cEEGrid (TMSi, Oldenzaal, Netherlands). This system has already proven useful in assessing inattentional deafness in ecological contexts (Dehais et al., 2019a). Pins on dry electrodes being sometimes painful when worn over long periods of time, solid gels were added to them in order to increase comfort (refer to Figure 1D), given the use of such technology has already proven useful for spectral analyses (Di Flumeri et al., 2019). In this study, the electrodes Fpz, Fz, Cz, Pz, Fp1, Fp2, C3, and C4 from the 10–20 standard system were mounted (Oostenveld and Praamstra, 2001) and referenced to the right and left earlobes as CMS and DRL derivations.

As recommended on the manufacturer's website, the participant's head and geltrodes were cleaned with glycerin before being positioned onto the cap on the participant's head. Once the neoprene cap was positioned on the participant's head, data were continuously and wirelessly recorded *via* Bluetooth connection at 500 Hz through the NIC2.0 software forwarding the data to the LSL Recorder.

##### 3.2.2.1. Pre-processing

To ensure a fair comparison, Enobio data were analyzed using a similar pipeline as cEEGrid data with the EEGLAB software (v.2019.1) (Delorme and Makeig, 2004) on MATLAB (The MathWorks, Natik, USA). Three main differences were to be observed: first, frontopolar electrodes were removed from the analysis, as they induced very bad results with the ASR correction; then, data were not re-referenced, as they were already referenced to the CMS and DRL electrodes; finally, no participants had to be removed from the analysis because of poor signal quality.

Similarly, raw EEG data were down-sampled to 250 Hz and band-pass filtered (Hamming window FIR, order: 414, 1 Hz transition bandwidth) between [1−20] Hz for ERPs and [1−40] Hz for frequency and time-frequency analyses. Data were denoised with ASR, interpolated, and cleaned with automated ICA (using the same parameters as for the cEEGrid processing). Finally, data were epoched time-locked to the sound stimuli (−200 to 1, 000 ms for ERPs, and −1 to 1.5 s for frequency data). Epoched data for ERPs were baseline corrected in the −200 to 0 ms time window before the stimulus.

One participant was removed from further processing due to technical failure from the recorder. The nine remaining participants were then averaged for each experimental condition separately according to the type of stimulus and response of the participant to target sounds (hits vs. misses). Usual ERPs and PSD were computed, as well as ERSPs, with the same parameters as for the cEEGrid data. In accordance with the literature on oddball paradigms (Luck and Kappenman, 2011), the Enobio ERP data were inspected for the N1 component at Cz around 100 ms post-stimulus (the most negative peak in the [50−150] ms post-stimulus time-window), and the P300 component at Pz starting around 300 ms post-stimulus onset (the average amplitude in the [300−500] ms post-stimulus time window). Similar to cEEGrid analyses, ERPs, PSDs, and ERSPs were compared for the odd sounds (hits vs. misses) at each time and/or frequency point with EEGLAB (Delorme and Makeig, 2004) through a permutation test with FDR correction for multiple comparisons. The statistical significance was set at α = 0.05.

### 3.3. Classification

Two different classifiers relying on Riemannian Geometry principles have been used: Minimum Distance to the Mean (MDM) classifier (Barachant et al., 2012) and classification in the Tangent Space using a logistic regression referred to as Tangent Space Classifier (TSC) (Barachant et al., 2012). These two classifiers, usually applied for active BCI experiments, use spatial covariance matrices of EEG signals as descriptors. The covariance matrices are semi-definite positive and, therefore, lie on a Riemannian Manifold (a subspace of the space of the *R*^*n*^ × *R*^*n*^ matrices) that has a specific geometry, a curvature. Riemmanian geometry principles ensure that the geometry of the manifold is taken into account, especially for distances computation. For instance, the Euclidean path between two points is a straight line that goes outside of the manifold, while Riemann distance, e.g., geodesic distance, is the shortest path that stays on the manifold (Appriou et al., 2020). MDM classifier computes a centroid, using geodesic distance, for each of the given classes. For a new test point, a class is affected according to the nearest centroid. On the other hand, TSC projects covariance matrices onto the tangent space of the manifold to produce features. These features are then fed to a logistic regression to perform classification. As demonstrated by a comparison of many algorithms (Appriou et al., 2020), these Riemannian Geometry Classifiers provide very competitive performances for passive BCI classification.

As advocated by Lotte (2015), shrinkage (or regularization) is used for the estimation of covariance matrices. This simple, computationally efficient, parameter-free method allows to reduce calibration time but also improve overall performance. In this study, we used the Schäfer-Strimmer shrinkage estimator (Schäfer and Strimmer, 2005) as it provides the best results with little training data. Additionally, data in the majority class were down-sampled using the classical Tomek Links procedure to ensure a balanced training dataset. Tomek Links procedure removes instances of the majority class that are the closest from samples of the opposite classes (Tomek, 1976). Finally, we applied a classical 5-fold cross-validation performance estimation.

To summarize, epochs previously extracted for the ERP analyses (−200 to 1, 000 ms around odd sounds stimulation; refer to section 3.2.1.1) were selected for the Enobio and cEEGrid recorded data. Covariance matrices with Schäfer-Strimmer shrinkage estimator were computed for each participant and data were down-sampled. Then, a 5-fold cross-validation procedure was applied on the Enobio (9 participants) and cEEGrid (8 participants) data using the MDM classifier and TSC. The results for each class accuracy (hits and misses), and the overall balanced accuracy of the classifier were computed.

Balanced classification accuracies were compared for each system separately (cEEGrid and Enobio) for the two classifiers (MDM vs. TSC) with pairwise *t*-test across participants.

## 4. Results

### 4.1. Behavioral Data

All participants managed to land safely on the runway in the two conditions.

There were on average the same number of oddball trials in the visibility conditions [279 ± 13 in the normal visibility and 286 ± 8 in the low visibility condition—*t*(9) = −0.86; *p* = 0.41], and the same odd/standard rate [75.5±0.65% standard in the normal visibility and 74.5 ± 0.32% in the low visibility condition—*t*(9) = 1.76; *p* = 0.11]. No significant difference was found in the miss rates between the normal visibility [33.1 ± 4.85%] and the low visibility [35.7 ± 6.48%; *t*(9) = −0.76; *p* = 0.47] conditions. Similarly, the sensitivity to sounds (*d*′) was equivalent in both conditions [*t*(9) = 1.75; *p* = 0.12], and was significantly greater than 0 in the normal visibility [3.2 ± 0.24, *t*(9) = 13.52, *p* < 1.10^−3^] and in the low visibility condition [2.7 ± 0.24, *t*(9) = 11.57, *p* < 1.10^−3^].

Concerning RT, there was no significant difference between RT to hits in the normal visibility [780.0±31.58 ms] and the low visibility [797.5±38.28 ms—*t*(9) = −0.76; *p* = 0.47] conditions.

**Figure 2:**
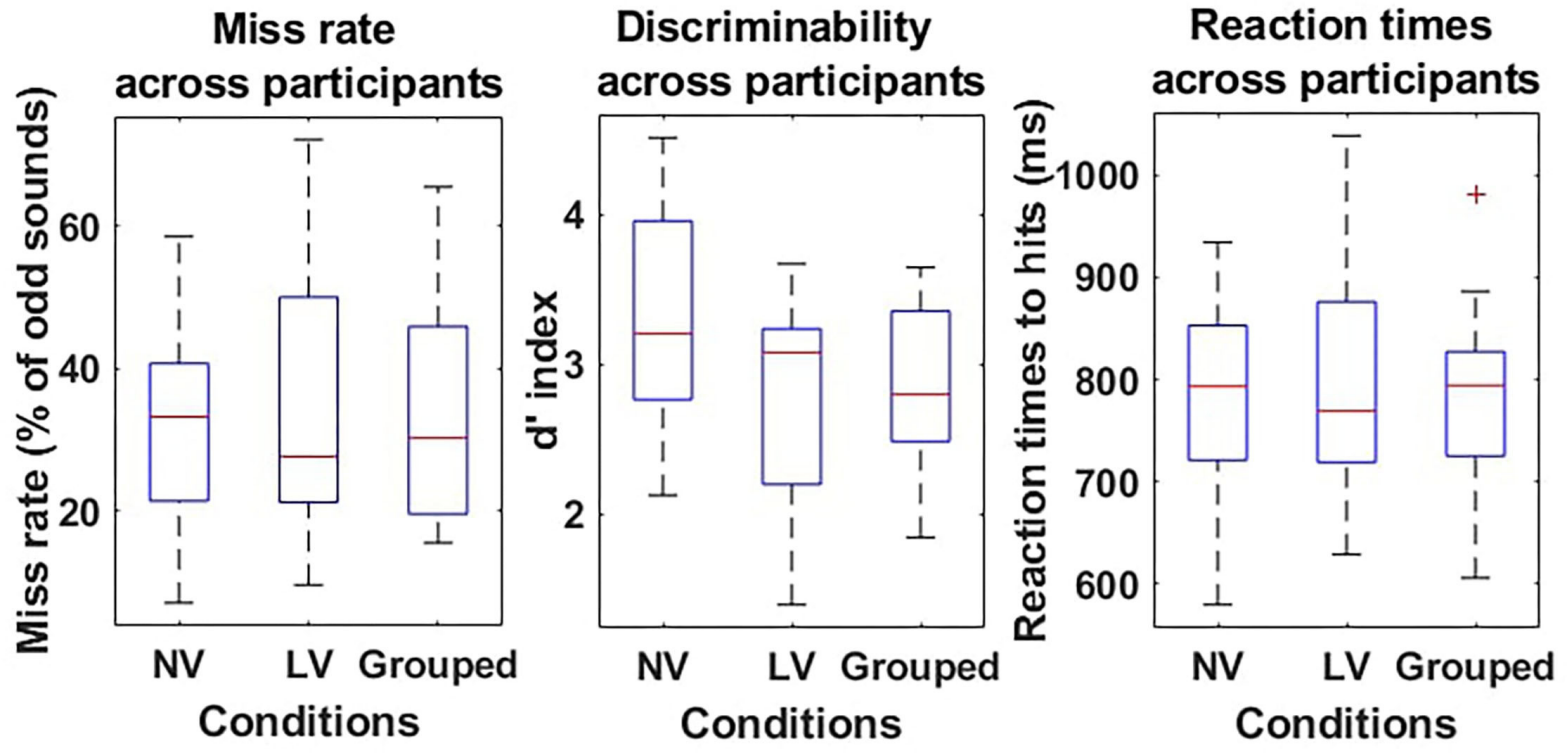
Behavioral results of the oddball task during flight simulation. Miss rates (%–left), discriminability *d*′ (a.u.–middle), and reaction times (RT) (ms–right) are presented as boxplots for the Normal visibility condition (NV–left-hand side boxplot), the low visibility condition (LV–middle boxplot), and the two conditions grouped (Grouped–right-hand side boxplot) for each measure. For each boxplot, the red line shows the median and the bottom and top of each box show the 25 and 75th percentile, respectively.

Given the absence of difference in the behavioral data, both runs were grouped for further analysis. Thus, the overall number of trials was 565±19 with 25 ± 0.41% target sounds. The global miss rate was 34.7 ± 5.43%, the false positive rate was 1.0±0.19%, the *d*′ was 2.8±0.19 and significantly different from 0 [*t*(9) = 15.14; *p* < 1.10^−3^], and the mean RT to hits was 789.1 ± 32.77 ms (Refer to Figure 2).

### 4.2. EEG Measures

#### 4.2.1. ERP Analysis

**Figure 3:**
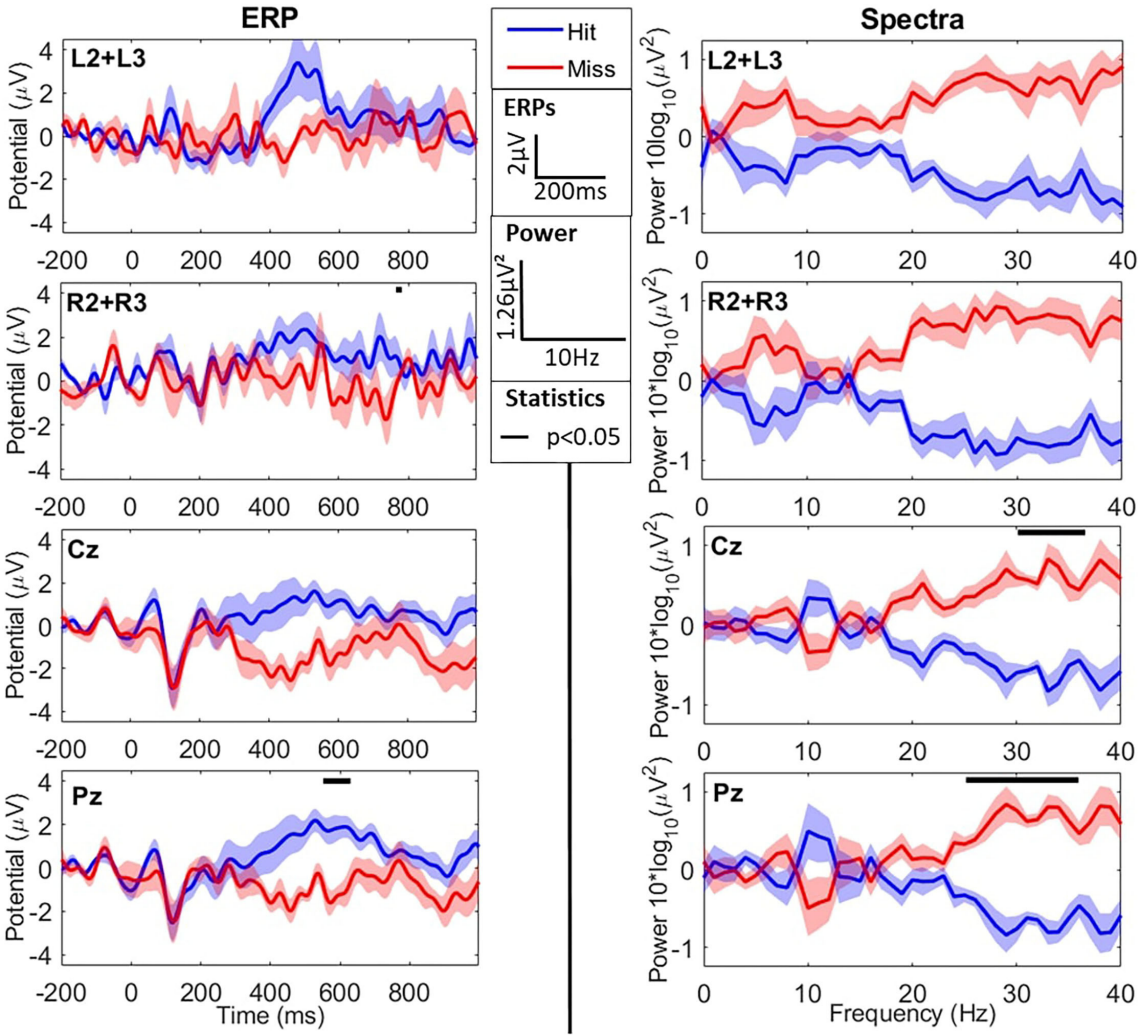
EEG results in the time (ERPs–left column) and frequency (Spectra–right column) domains for the oddball task during flight simulation. The two first lines show cEEGrid data, whereas the two last ones show Enobio data. Data are averaged across difficulty levels (refer to sections 3.2.1.1 and 3.2.2.1) and show grand averages across participants for hits (blue) and misses (red). ERPs are averaged for each type of trial from −200 ms to 1 s around sound presentation (*t* = 0). Power Spectral Densities (PSD) are presented with individual means subtracted from spectra. From first to last, lines show the averages at the left grid L2+L3 electrodes, the right grid R2+R3 electrodes, Cz electrode, and Pz electrode. Black lines at the top of graphs show windows (in time or frequency) where averages for hits and misses are significantly different. ERPs (in μ*V*) and spectra (as 10 × *log*_10_(*PSD*) in μ*V*^2^) are presented as mean±SD across participants.

When comparing the two types of stimuli (hits vs. misses), we observed an N1 component for both peaking around 200 ms post-stimulus for the cEEGrid and slightly earlier (~100 ms for the Enobio data (consistently with the literature—refer to Figure 3 and Table 2). Similarly, we observed a later and broad P300 component only for hits, with both the cEEGrid and Enobio systems. Consistently with the literature, no P300 was observed for misses. Permutation tests revealed a significant difference between the two conditions at the Pz electrode in a [500−572] ms time window. A significant difference was also observed between 712 and 716 ms post-stimulus on the right-sided cEEGrid electrodes.

**Table 2:** Average ± SEM amplitude of the N1 (top part) and P300 (bottom port) recorded with the cEEGrid (left hand-side) around the left ear (first column–an average of L2 and L3 electrodes) and the right ear (second column–an average of R2 and R3 electrodes), and with the solid gel-based Enobio at the Cz (third column) and Pz (last column) electrodes.

		**cEEGrid**		**Enobio**	
		**L2+L3**	**R2+R3**	**Cz**	**Pz**
N1	Hits	−1.51 ± 0.38 μ*V*	−1.04 ± 0.54 μ*V*	−2.00 ± 0.95 μ*V*	−1.69 ± 0.85 μ*V*
		at 171 ± 4.55 ms	at 174 ± 5.71 ms	at 106.44 ± 9.71 ms	at 104.22 ± 9.36 ms
	Misses	−0.71 ± 0.79 μ*V*	−1.95 ± 0.93 μ*V*	−2.93 ± 1.14 μ*V*	−2.72 ± 1.04 μ*V*
		at 196 ± 6.36 ms	at 191.5 ± 8.00 ms	at 108.67 ± 10.44 ms	at 113.11 ± 11.40 ms
P300	Hits	1.14 ± 0.77 μ*V*	1.57 ± 0.59 μ*V*	1.12 ± 0.71 μ*V*	1.47 ± 0.57 μ*V*
	Misses	−0.26 ± 0.36 μ*V*	0.36 ± 0.63 μ*V*	−1.78 ± 0.71 μ*V*	−1.06 ± 0.28 μ*V*

*N1 amplitude is computed at its peak latency for hits (first line) and misses (second line). P300 amplitude is computed as the average amplitude in the [300−500] ms post odd sound presentation time-window for hits (third line) and misses (last line)*.

Even though these data are consistent with the literature, the amplitude of the N1 and P300 are lower than with the usual cap EEG. The peak N1 amplitudes and latencies, as well as average P300 amplitudes for cEEGrid and Enobio data, are reported in Table 2.

#### 4.2.2. Spectral Analysis

Spectral activity in the two conditions (hits vs. misses) is presented in Figure 3. Permutation analysis on cEEGrid data revealed a tendency for an effect of the type of stimulus (hit vs. miss; refer to Figure 3) on high-frequency spectral activity in the β and γ bands: [20−40] Hz frequency windows for both the right and left grid electrodes (*ps* = 0.062). In addition, the same tendency was observed in the α frequency band ([7−8] Hz) for the left-sided electrodes (L2+L3) only.

Concerning the Enobio data, permutation tests revealed a significant effect of the stimulus-response condition in the high β/low γ frequency bands (>25 Hz—refer to Figure 3). This difference was more consistent at the Pz electrode but also observed at Cz. The average power in the high β band ([25−30] Hz) was significantly lower for hits compared to misses at Pz (−4.14 ± 0.66 μ*V*^2^ vs. −3.02 ± 0.62 μ*V*^2^, respectively—*p* < 0.015) electrode. Similarly, the average power in the low γ band ([30−40] Hz) was significantly lower for hits compared to misses at Cz (−3.35 ± 0.60 μ*V*^2^ vs. −5.04 ± 0.67 μ*V*^2^, respectively—*p* < 0.03) and Pz (−5.20 ± 0.75 μ*V*^2^ vs. −3.73 ± 0.74 μ*V*^2^, respectively—*p* < 0.015) electrodes.

#### 4.2.3. Time-Frequency Analysis

**Figure 4:**
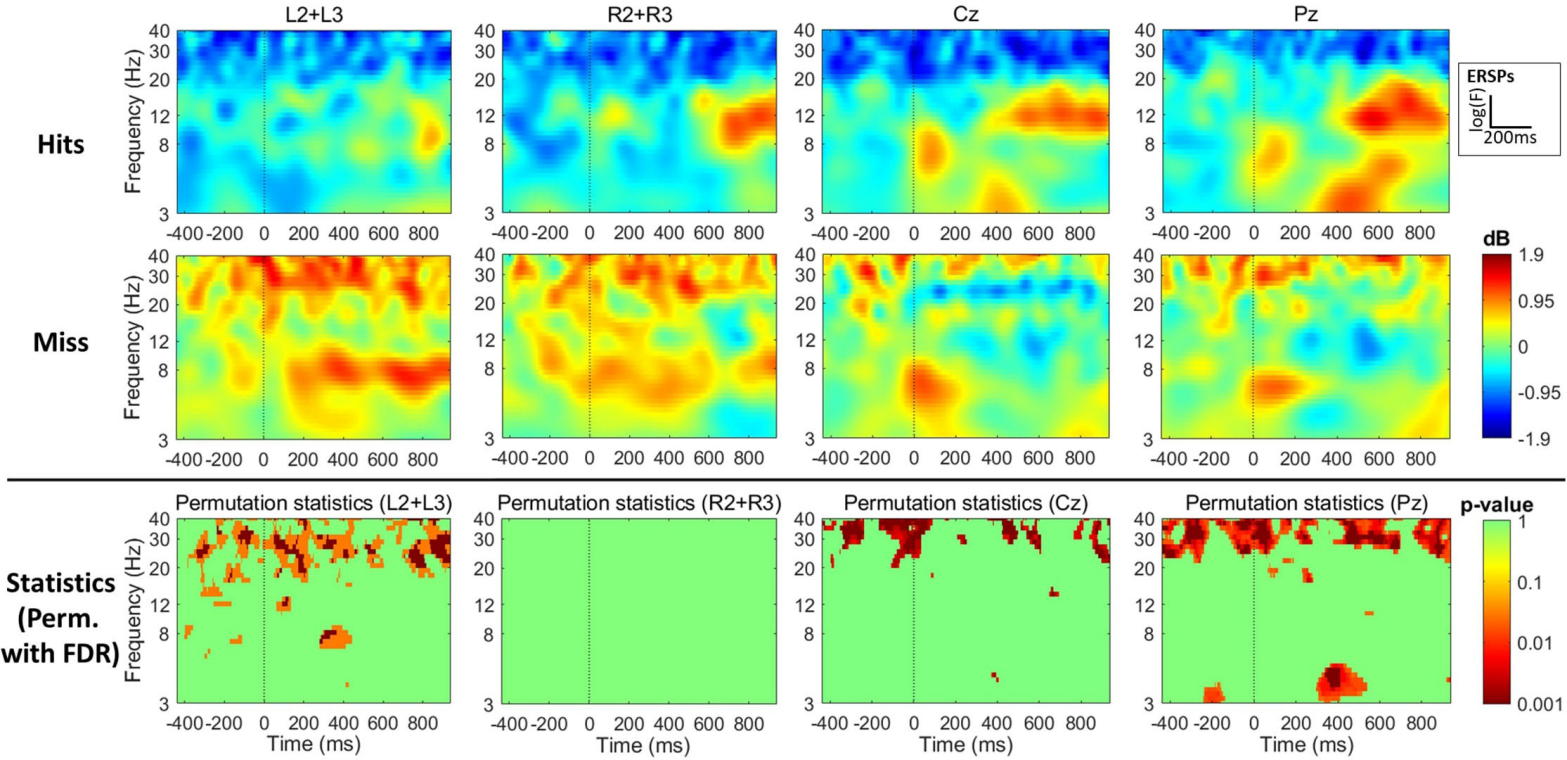
EEG results in the time-frequency domain for the oddball task during flight simulation. Data for hits (top line) and misses (middle line), as well as the permutation statistics (FDR correction for multiple comparisons) between the two conditions, are shown for the cEEGrid recording at the left hear (average of L2 and L3–first column) and the right hear (average of R2 and R3–second column), and the Enobio recording at Cz (third column) and Pz (fourth column) electrodes. All data (except statistics) are presented as the average power (in dB) increase (red data) or decrease (blue data) relative to baseline with a common baseline across conditions (hits vs. miss) for each electrode or electrode average. Statistics (third line) show the statistical significance as obtained with the *p* − *value* revealing a significant difference (red) or not (green) between the compared conditions (hits vs. misses). The x-axis shows the time course of data across the 250 time-points selected in a −442 to 938 ms time-window centered on stimulus display (*t* = 0). The y-axis shows the frequency range (log-scale between 3 and 40 Hz) across which 40 frequencies were selected for wavelet decomposition.

Event-Related Spectral Perturbations revealed a general increase in power, relative to baseline, for hits and a general decrease for misses. In addition, as observed through spectral analyses, there is a global higher activity in the high β/low γ ([20−40] Hz) frequency bands for misses compared to hits, as revealed by the permutation test between the two conditions (refer to Figure 4) and their statistics. In addition, we can observe on left-sided electrodes, a significant difference between the two conditions in the θ frequency band ([4−8] Hz) between 260 and 460 ms post-stimulus.

Concerning the Enobio data, the ERSPs also revealed a general increase in power relative to baseline for hits, and a general decrease for misses. In addition, and similarly to cEEGrid data, there is a global increase in the high β/low γ frequency bands ([20−40] Hz) throughout the whole trial. There is, thus, an effect of the type of trial (hits vs. misses) on the high frequency bands (refer to Figure 4). In addition, and once again consistent with the cEEGrid data, we observed a significant effect of the type of trial on time-frequency measures in the θ band ([4−8] Hz) in the 297−546 ms time-window. Nevertheless, it has to be pointed that, in this study, we observed a decrease of activity for misses compared to hits, and that this decrease appears in the low θ frequencies (as compared to high θ for cEEGrid data).

### 4.3. Classification

**Figure 5:**
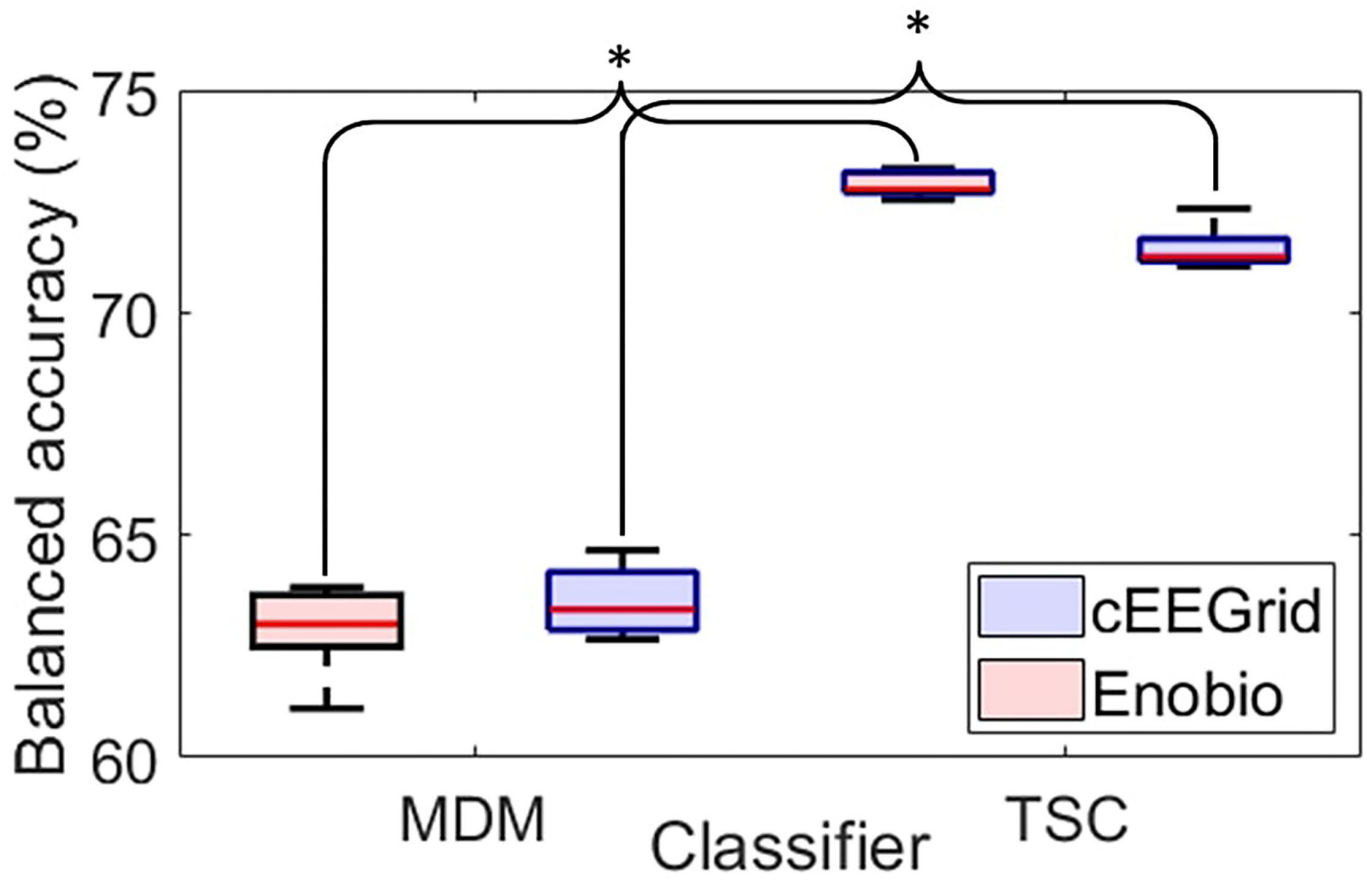
Boxplots of the balanced accuracy distribution over the 5-folds cross-validation for the Minimum Distance to the Mean classifier (MDM–left-hand side) and the Tangent Space Classifier (TSC–right-hand side) for the cEEGrid (blue) and the Enobio (red) data. For each box, the central red line corresponds to the median, the top and bottom edges indicate the 25 and 75*th* percentiles, and the whiskers extend to the most extreme data points not considered outliers. *p < 0.05.

The distribution of classification accuracy over the five cross-validation steps is presented for both classifiers and each EEG system in Figure 5.

The MDM classifier (left-hand side of Figure 5) revealed an average accuracy of 63.52 ± 0.41% to classify cEEGrids ERPs and of 62.89 ± 0.53% to classify Enobio ERPs with 5-fold cross-validation.

Tangent space classification (right-hand side of Figure 5) revealed an average accuracy of 71.45 ± 0.15% to classify cEEGrid ERPs and of 72.88 ± 0.26% to classify Enobio ERPs with 5-fold cross-validation.

Pairwise *t*-tests revealed significantly better classification accuracies with the TSC compared to MDM for the cEEGrid [*t*(7) = −3.31;*p* < 0.05] and the Enobio [*t*(8) = −2.77;*p* < 0.05] systems.

## 5. Discussion

Recent technological advances in the field of highly portable neurophysiological sensors offer interesting perspectives to study brain functioning in the real world (Fairclough and Lotte, 2020; Gramann et al., 2021). The main motivation of this study was to benchmark two non-invasive and comfortable ultra-mobile EEG systems to study inattentional deafness-related brain activities in a motion flight simulator. First, an ear-centered 10-flex-printed-electrode device (i.e., the cEEGrid); second an adapted drytrode Enobio system on which solid gels were inserted to prevent discomfort on the scalp. Participants had to perform two approaches and final landings along with an auditory oddball task. These short but intense flight phases were chosen since previous studies indicated that they are prone to promote inattentional deafness (Dehais et al., 2019c). The two approaches had varying visibility (low and normal) to induce variation and avoid the habituation effect. Indeed, the repetition of these two scenarios allowed us to maximize the number of episodes of inattentional deafness to improve the SNR of our electrophysiological analyses (i.e., ERPs). During these two runs, brain activity was recorded continuously and concurrently with the two systems and analyzed in the time (ERPs), frequency (PSDs), and time-frequency (ERSPs) domains. A further single trial classification was then performed. These measures were used as objective measures to benchmark these two EEG systems.

The behavioral results showed the efficiency of the two scenarios to promote, high rate of auditory misses (i.e., ~35% compared to the 2% miss rate across laboratories in an inter-lab experiment—Alexander et al., 1994). The miss rate, mean reaction time, and discriminability (*d*′) were identical across the two scenarios which both involved final approach and landing that are known to be particularly demanding and engaging (i.e., they involve the supervision of flight instruments to reach a final destination; Dehais et al., 2019c). This similarity is consistent with our expectation as these instrument-based landings do not require outside visibility except at the very last minute for approach and landing on the runway. Also, the average discriminability values across the two runs (mean *d*′ = 2.8 ± 0.19>0) demonstrates that the failure to detect alarms is not due to more difficulties to identify the auditory stimuli, but evidently to the challenging flying task. This finding, together with others (Durantin et al., 2017; Callan et al., 2018; Dehais et al., 2019b,c), confirms the importance of conducting neuroergonomics experiments in ecological settings to investigate such complex phenomenon rather than in simplified laboratory settings. Basic experiments generally fail to induce such lapse in auditory attention thus solely reporting the effects of load on auditory processing (Molloy et al., 2015).

Time-domain analyses with the cEEGrid did not allow us to identify differences in amplitude of the N100 and P300 between hits and misses as indicated by Callan et al. (2018) with a 64 dry EEG system, Dehais et al. (2019b) with a 32 dry EEG system and by Dehais et al. (2019c) with a 32 wet research grad EEG system. Though one could observe the N1-like ERP peaking around 200 ms on the right-ear electrodes, as well as a P300-like ERP on the left ear ones, these results did not pass the significance threshold. Previous cEEGrid studies (Hölle et al., 2021) successfully reported differences in P300 amplitude during an oddball task out of the lab. However, in our case, we used a motion flight simulator that is known to induce several motion artifacts or electromagnetic perturbations: eye movements to scan the environment, muscular activity to handle the stick and rudder, electrical/electromagnetic interference due to several computers and screens. This noise may have strongly affected this system that is not pre-amplified and in return prevented us from identifying classical electrophysiological correlates of inattentional deafness. Possibly, a larger number of subjects may counterbalance artifacts and perturbations to allow the results to pass the significance threshold. Nevertheless, the time domain analyses computed over the data collected with the Enobio system disclosed that the P300 amplitude was significantly reduced for misses compared to hits on Pz electrode similarly to Giraudet et al. (2015), Callan et al. (2018), and Dehais et al. (2019b,c). Interestingly, the latency of measure of the P300 as determined by the permutation test is slightly increased compared to lab-recorded P300 (roughly 300−500 ms). Yet, it is consistent with other studies in highly demanding flight simulation and in-flight conditions (Giraudet et al., 2015; Dehais et al., 2019a) revealing later (400−700 or 500−750 ms post-stimulus) and broader P300. This is also consistent with theories on the P300 disclosing an effect of stimulus evaluation processes and, thus, the quantity of noise surrounding the stimuli, on the P300 latency (Magliero et al., 1984). On the other hand, no differences were found for the N100 on Cz or Fz electrodes as one could expect. Again the environment of the flight simulator as well as the limited number of electrodes, preventing us from performing advanced processing (e.g., cleaner ICA decomposition with an increased number of components), may explain this lack of results.

The frequency domain (PSD) analysis also did not lead to statistical differences with the cEEGrid when comparing auditory misses to hits. However, the finding with the Enobio indicated the usability of the dry-electrodes associated with the solid gel for PSD decomposition across as revealed by higher spectral β and γ power for hits compared to misses. Increased β band activity is generally associated with high arousal states (Okogbaa et al., 1994) and were found to account for the pilot's mental overload (Dehais et al., 2020b). Similarly, higher γ band power is thought to reflect brain response to higher task demand (Fitzgibbon et al., 2004). These results, together with our behavioral findings, confirm that high load can induce inattentional deafness. It has to be noted though, that we cannot exclude an effect of muscular activity reflected in the high-frequency (>20 Hz) difference between hits and misses. Indeed, it has been shown that brain activity above 20 Hz recorded during cognitive task execution could be contaminated by muscular activity (Whitham et al., 2007). However, in this study, the time-frequency plots seems to indicate the absence of muscular artifact, as they demonstrate a higher activity throughout the entire trial, not only in relation to the moment of the response. To the author's knowledge, only one study had previously assessed spectral-domain activity quantification with solid gel (Di Flumeri et al., 2019), however, their analysis was centered on the variations of mean power in specific frequency bands across time, and they neither displayed the whole PSD across frequencies nor time-domain characteristics. We ran more advanced analyses such as the study of time-frequency evoked responses with the two systems. The Enobio results disclosed a decreased power in the δ/low θ band for misses compared to auditory hits. The δ brain waves are thought to play an important role in synchronizing different brain areas for optimal performance. Nevertheless, these synchronization/desynchronization (ERS/ERD) patterns seem to be inverted for low frequency bands (<8 Hz) between the Enobio and the cEEGrid system preventing us from drawing clear outputs on time-frequency measures. Indeed, the Enobio demonstrated a significant decrease in δ/low θ frequency bands for misses compared to hits at the Pz electrode. Ponjavic-Conte et al. (2012) reported lower activity in the θ band during auditory attention distraction, but they only inspected the Cz electrode. The activity of the low frequencies has also been implicated on numerous occasions in auditory perceptual sampling processes (Kubetschek and Kayser, 2021). Finally, we observe both at Cz and Pz electrodes, like at the left grid of the cEEGrid, increased β and θ power spectral variations for misses compared to hits. These variations being very consistent throughout the entire trial (in the time domain) are most likely a representation of the mental overload or at least very high mental demand, which could be responsible for auditory misses.

Not only were the results demonstrable with both EEG recording systems, but also *i)* with only a small number of participants, and *ii)* with very fast and automated pre-processing. Relevant activity was extracted from only 8 participants for the cEEGrid and 9 participants for the Enobio system. The fact that we were able to recognize ERPs and spectral activity with a complex, skill-requiring task is very promising for the neuroergonomics field. In this field, and especially in aviation, it is very common to perform experiments on trained expert populations possessing specific skills. It can, thus, be difficult to recruit participants, and more precisely in the same amount as is usually done in the cognitive neuroscience area. Nevertheless, an increasing number of studies are demonstrating the feasibility of small sample analyses (Zander et al., 2017; Hölle et al., 2021), with classification accuracies and data equivalent to ours. In this study, we managed to ensure a reasonable classification accuracy for inattentional deafness brain correlates. The obtained accuracies with the TSC classifier, which performed best, are in the same range as the one observed for auditory attention measures with the cEEGrid and a cap-EEG (Bleichner et al., 2016) but also classification studies on research-grade 32-active electrodes cap-EEG data for inattentional deafness detection during flight simulation (Dehais et al., 2019c). Interestingly, these considerations go beyond the scope of Neuroergonomics toward the Brain Computer Interface (BCI) community where more and more research is centered on transfer learning (i.e., between participant, task, session validity of measures, and algorithms) among others in order to compensate for a small number of patients or participants in studies (Wan et al., 2021). Finally, the two systems used in this study were comfortable and required only a small amount (cEEGrid) to no gel (Enobio) at all. Removing the barriers of both the gel and the number of electrodes opens the perspective of helmet-mounted EEG systems for *in situ* measures of operators' mental state with maximum transparency of the recording device.

In summary, in this study, we managed to obtain more than 70% of accuracy to classify inattentional deafness on a small pool of expert participants in a neuroergonomics applied context, with two unobtrusive, comfortable and mobile EEG systems, paving the way for more out-of-the-lab, or even operational studies of cognitive processes and difficulties. Further studies need to be done though to evaluate the stability of both cEEGrid (TMSi, Oldenzaal, Netherlands) and Enobio (Neuroelectrics, Barcelona, Spain) signals over long periods of time as the cEEGrid might loosen and detach from the skin, as much as the solid gel might become rigid with time which in return could attenuate the quality of the signal.

## Data Availability Statement

The datasets presented in this study can be found in online repositories. The names of the repository/repositories and accession number(s) can be found below: OSF repository: Inattentional deafness in flight simulation (http://www.doi.org/10.17605/OSF.IO/C2YG4).

## Ethics Statement

The studies involving human participants were reviewed and approved by the Institutional Review Board of the Comité d'Evaluation Ethique de l'Inserm (IRB00003888-18-460). The patients/participants provided their written informed consent to participate in this study.

## Author Contributions

BS and FD contributed to the conception and design of the study. BS and YG recorded the data and performed part of the analyses. LD developed the classification pipelines and performed the machine learning analyses. BS wrote the first draft of the manuscript. All authors revised and rewrote parts of the manuscript, read, and approved the submitted version.

## Funding

This research was funded by the Agence Innovation Défense (AID) of the Direction Générale de l'Armement on the MAIA (Modelling Attention for Adaptative Interaction) project.

## Conflict of Interest

The authors declare that the research was conducted in the absence of any commercial or financial relationships that could be construed as a potential conflict of interest.

## Publisher's Note

All claims expressed in this article are solely those of the authors and do not necessarily represent those of their affiliated organizations, or those of the publisher, the editors and the reviewers. Any product that may be evaluated in this article, or claim that may be made by its manufacturer, is not guaranteed or endorsed by the publisher.

## References

[B1] Alexander J. E. Polich J. Bloom F. E. Bauer L. O. Kuperman S. Rohrbaugh J.. (1994). P300 from an auditory oddball task: inter-laboratory consistency. Int. J. Psychophysiol. 17, 35–46. 10.1016/0167-8760(94)90053-1 7961052

[B2] Anderson N. D. (2015). Teaching signal detection theory with pseudoscience. Front. Psychol. 6:762. 10.3389/fpsyg.2015.00762 26089813 PMC4452803

[B3] Appriou A. Cichocki A. Lotte F. (2020). Modern machine-learning algorithms: For classifying cognitive and affective states from electroencephalography signals. IEEE Syst. Man Cybern. Mag. 6, 29–38. 10.1109/MSMC.2020.296863827295638

[B4] Artieda J. Valencia M. Alegre M. Olaziregi O. Urrestarazu E. Iriarte J. (2004). Potentials evoked by chirp-modulated tones: a new technique to evaluate oscillatory activity in the auditory pathway. Clin. Neurophysiol. 115, 699–709. 10.1016/j.clinph.2003.10.021 15036066

[B5] Barachant A. Bonnet S. Congedo M. Jutten C. (2012). Multiclass brain computer interface classification by riemannian geometry. IEEE Trans. Biomed. Eng. 59, 920–928. 10.1109/TBME.2011.2172210 22010143

[B6] Bleichner M. G. Debener S. (2019). Independent component decomposition of around ear EEG data to detect artifacts, in 2019 IEEE International Conference on Systems, Man and Cybernetics (SMC) (Bari: IEEE), 3631–3634.

[B7] Bleichner M. G. Mirkovic B. Debener S. (2016). Identifying auditory attention with ear-EEG: cEEGrid versus high-density cap-EEG comparison. J. Neural Eng. 13, 066004. 10.1088/1741-2560/13/6/066004 27705963

[B8] Blum S. Mirkovic B. Debener S. (2019). Evaluation of riemannian ASR on cEEGrid data: an artifact correction method for bcis, in 2019 IEEE International Conference on Systems, Man and Cybernetics (SMC) (Bari: IEEE), 3625–3630.

[B9] Borghini G. Astolfi L. Vecchiato G. Mattia D. Babiloni F. (2014). Measuring neurophysiological signals in aircraft pilots and car drivers for the assessment of mental workload, fatigue and drowsiness. Neurosci. Biobehav. Rev. 44, 58–75. 10.1016/j.neubiorev.2012.10.003 23116991

[B10] Brainard D. H. (1997). The psychophysics toolbox. Spat Vis. 10, 433–436. 10.1163/156856897X00357 9176952

[B11] Callan D. E. Gateau T. Durantin G. Gonthier N. Dehais F. (2018). Disruption in neural phase synchrony is related to identification of inattentional deafness in real-world setting. Hum. Brain Mapp. 39, 2596–2608. 10.1002/hbm.24026 29484760 PMC6866488

[B12] Campagne A. Pebayle T. Muzet A. (2004). Correlation between driving errors and vigilance level: influence of the driver's age. Physiol. Behav. 80, 515–524. 10.1016/j.physbeh.2003.10.004 14741236

[B13] cEEGrid-Stefan Debener (2019). cEEGrid-How to Use. Available online at: https://uol.de/neuropsychologie/howtouse

[B14] Chang C.-Y. Hsu S.-H. Pion-Tonachini L. Jung T.-P. (2018). Evaluation of artifact subspace reconstruction for automatic EEG artifact removal, in Conference Proceedings: Annual International Conference of the IEEE Engineering in Medicine and Biology Society. IEEE Engineering in Medicine and Biology Society. Conference (Honolulu, HI), 1242–1245. 10.1109/EMBC.2018.8512547 30440615

[B15] Chang C.-Y. Hsu S.-H. Pion-Tonachini L. Jung T.-P. (2019). Evaluation of artifact subspace reconstruction for automatic artifact components removal in multi-channel EEG Recordings. IEEE Trans. Biomed. Eng. 67, 1114–1121. 10.1109/EMBC.2018.8512547 31329105

[B16] Craig A. Tran Y. Wijesuriya N. Nguyen H. (2012). Regional brain wave activity changes associated with fatigue. Psychophysiology 49, 574–582. 10.1111/j.1469-8986.2011.01329.x 22324302

[B17] Dalton P. Fraenkel N. (2012). Gorillas we have missed: sustained inattentional deafness for dynamic events. Cognition 124, 367–372. 10.1016/j.cognition.2012.05.012 22726569

[B18] Debener S. Emkes R. De Vos M. Bleichner M. (2015). Unobtrusive ambulatory EEG using a smartphone and flexible printed electrodes around the ear. Sci. Rep. 5:16743. 10.1038/srep16743 26572314 PMC4648079

[B19] Dehais F. Duprès A. Blum S. Drougard N. Scannella S. Roy R. N.. (2019a). Monitoring pilot's mental workload using erps and spectral power with a six-dry-electrode EEG system in real flight conditions. Sensors 19, 1324. 10.3390/s19061324 30884825 PMC6471557

[B20] Dehais F. Lafont A. Roy R. Fairclough S. (2020a). A neuroergonomics approach to mental workload, engagement and human performance. Front. Neurosci. 14:268. 10.3389/fnins.2020.00268 32317914 PMC7154497

[B21] Dehais F. Rida I. Roy R. N. Iversen J. Mullen T. Callan D. (2019b). A pbci to predict attentional error before it happens in real flight conditions, in 2019 IEEE International Conference on Systems, Man and Cybernetics (SMC) (Bari: IEEE), 4155–4160.

[B22] Dehais F. Roy R. N. Scannella S. (2019c). Inattentional deafness to auditory alarms: Inter-individual differences, electrophysiological signature and single trial classification. Behav. Brain Res. 360, 51–59. 10.1016/j.bbr.2018.11.045 30508609

[B23] Dehais F. Somon B. Mullen T. Callan D. E. (2020b). A neuroergonomics approach to measure pilot's cognitive incapacitation in the real world with eeg, in International Conference on Applied Human Factors and Ergonomics (Virtual Conference, USA: Springer).

[B24] Delorme A. Makeig S. (2004). EEGLAB: an open source toolbox for analysis of single-trial EEG dynamics including independent component analysis. J. Neurosci. Methods 134, 9–21. 10.1016/j.jneumeth.2003.10.009 15102499

[B25] Di Flumeri G. Aricò P. Borghini G. Sciaraffa N. Di Florio A. Babiloni F. (2019). The dry revolution: evaluation of three different EEG dry electrode types in terms of signal spectral features, mental states classification and usability. Sensors 19, 1365. 10.3390/s19061365 30893791 PMC6470960

[B26] Durantin G. Dehais F. Gonthier N. Terzibas C. Callan D. E. (2017). Neural signature of inattentional deafness. Hum. Brain Mapp. 38, 5440–5455. 10.1002/hbm.23735 28744950 PMC6866714

[B27] Fairclough S. H. Lotte F. (2020). Grand challenges in neurotechnology and system neuroergonomics. Front. Neuroergonomics 1:2. 10.3389/fnrgo.2020.602504 PMC10790858 38234311

[B28] Fitzgibbon S. Pope K. Mackenzie L. Clark C. Willoughby J. (2004). Cognitive tasks augment gamma EEG power. Clin. Neurophysiol. 115, 1802–1809. 10.1016/j.clinph.2004.03.009 15261859

[B29] Giraudet L. St-Louis M.-E. Scannella S. Causse M. (2015). P300 event-related potential as an indicator of inattentional deafness? PLoS ONE 10:e0118556. 10.1371/journal.pone.0118556 25714746 PMC4340620

[B30] Gramann K. McKendrick R. Baldwin C. Roy R. N. Jeunet C. Mehta R. K.. (2021). Grand field challenges for cognitive neuroergonomics in the coming decade. Front. Neuroergonomics 2:6. 10.3389/fnrgo.2021.643969 PMC10790834 38235233

[B31] Hettinger L. J. Branco P. Encarnacao L. M. Bonato P. (2003). Neuroadaptive technologies: applying neuroergonomics to the design of advanced interfaces. Theor. Issues Ergon. Sci. 4, 220–237. 10.1080/1463922021000020918

[B32] Hölle D. Meekes J. Bleichner M. G. (2021). Mobile ear-eeg to study auditory attention in everyday life. Behav. Res. Methods 53, 2025–2036. 10.3758/s13428-021-01538-0 33721208 PMC8516794

[B33] Jasper H. H. (1958). The ten-twenty electrode system of the international federation. Electroencephalogr. Clin. Neurophysiol. 10, 370–375. 10590970

[B34] Justen C. Herbert C. (2018). The spatio-temporal dynamics of deviance and target detection in the passive and active auditory oddball paradigm: a sLORETA study. BMC Neurosci. 19:25. 10.1186/s12868-018-0422-3 29673322 PMC5909247

[B35] Kleiner M. Brainard D. Pelli D. (2007). What's new in psychtoolbox-3. Perception 36, 1–16.

[B36] Kothe C. Medine D. Boulay C. Grivich M. Stenner T. (2019). LSL-The LabStreaming Layer. Available online at: https://github.com/sccn/labstreaminglayer

[B37] Kubetschek C. Kayser C. (2021). Delta/theta band EEG activity shapes the rhythmic perceptual sampling of auditory scenes. Sci. Rep. 11, 1–15. 10.1038/s41598-021-82008-7 33504860 PMC7840678

[B38] Lotte F. (2015). Signal processing approaches to minimize or suppress calibration time in oscillatory activity-based brain-computer interfaces. Proc. IEEE 103, 871–890. 10.1109/JPROC.2015.240494127295638

[B39] Luck S. J. Kappenman E. S. (2011). The Oxford Handbook of Event-Related Potential Components. New York, NY: Oxford University Press.

[B40] Macdonald J. S. Lavie N. (2011). Visual perceptual load induces inattentional deafness. Attent. Percept. Psychophys. 73, 1780–1789. 10.3758/s13414-011-0144-4 21611856 PMC3152714

[B41] Magliero A. Bashore T. R. Coles M. G. Donchin E. (1984). On the dependence of p300 latency on stimulus evaluation processes. Psychophysiology 21, 171–186. 10.1111/j.1469-8986.1984.tb00201.x 6728983

[B42] Makeig S. Debener S. Onton J. Delorme A. (2004). Mining event-related brain dynamics. Trends Cogn. Sci. 8, 204–210. 10.1016/j.tics.2004.03.008 15120678

[B43] Martin G. Bleichner. (2019). cEEGrid Plugin. Available online at: https://gitlab.com/mgbleichner/ceegridplugin

[B44] Mirkovic B. Bleichner M. G. De Vos M. Debener S. (2016). Target speaker detection with concealed EEG around the ear. Front. Neurosci. 10:349. 10.3389/fnins.2016.00349 27512364 PMC4961688

[B45] Miyakoshi M. Kothe C. (2019). Artifact Subspace Reconstruction Plug-in. Available online at: https://github.com/sccn/clean_rawdata

[B46] Molloy K. Griffiths T. D. Chait M. Lavie N. (2015). Inattentional deafness: visual load leads to time-specific suppression of auditory evoked responses. J. Neurosci. 35, 16046–16054. 10.1523/JNEUROSCI.2931-15.2015 26658858 PMC4682776

[B47] Näätänen R. Picton T. (1987). The n1 wave of the human electric and magnetic response to sound: a review and an analysis of the component structure. Psychophysiology 24, 375–425. 10.1111/j.1469-8986.1987.tb00311.x 3615753

[B48] Okogbaa O. G. Shell R. L. Filipusic D. (1994). On the investigation of the neurophysiological correlates of knowledge worker mental fatigue using the EEG signal. Appl. Ergon. 25, 355–365. 10.1016/0003-6870(94)90054-X 15676987

[B49] Oostenveld R. Praamstra P. (2001). The five percent electrode system for high-resolution EEG and ERP measurements. Clin. Neurophysiol. 112, 713–719. 10.1016/S1388-2457(00)00527-7 11275545

[B50] Pacharra M. Debener S. Wascher E. (2017). Concealed around-the-ear EEG captures cognitive processing in a visual simon task. Front. Hum. Neurosci. 11:290. 10.3389/fnhum.2017.00290 28642695 PMC5462961

[B51] Parasuraman R. (2003). Neuroergonomics: Research and practice. Theor. Issues Ergon. Sci. 4, 5–20. 10.1080/14639220210199753

[B52] Pion-Tonachini L. Kreutz-Delgado K. Makeig S. (2019). ICLabel: An automated electroencephalographic independent component classifier, dataset, and website. Neuroimage 198, 181–197. 10.1016/j.neuroimage.2019.05.026 31103785 PMC6592775

[B53] Ponjavic-Conte K. D. Dowdall J. R. Hambrook D. A. Luczak A. Tata M. S. (2012). Neural correlates of auditory distraction revealed in theta-band EEG. Neuroreport 23, 240–245. 10.1097/WNR.0b013e3283505ac6 22314684

[B54] Poussot-Vassal C. Roy R. N. Bovo A. Gateau T. Dehais F. Ponzoni Carvalho Chanel C. (2017). A loewner-based approach for the approximation of engagement-related neurophysiological features, in Proceedings of the International Federation of Automatic Control (Toulouse).

[B55] Schäfer J. Strimmer K. (2005). A shrinkage approach to large-scale covariance matrix estimation and implications for functional genomics. Stat. Appl. Genet. Mol. Biol. 4:32. 10.2202/1544-6115.1175 16646851

[B56] Segalowitz S. J. Barnes K. L. (1993). The reliability of ERP components in the auditory oddball paradigm. Psychophysiology 30, 451–459. 10.1111/j.1469-8986.1993.tb02068.x 8416071

[B57] Somon B. Roy R. N. Simonetti I. Dehais F. (2021). Ecological measures of cognitive impairments in aeronautics: theory and application, in Current Research in Neuroadaptive Technology, Chapter 7, eds Zander T. O. Fairclough S. (San Diego, CA: Elsevier). p. 117–138. 10.1016/B978-0-12-821413-8.00012-9

[B58] Somon B. Simonetti I. Roy R. N. Dehais F. (2019). Apprehending auditory activity in ecological contexts with unobtrusive EEG, in The Second Neuroadaptive Technology Conference (Liverpool), 72.

[B59] Sterr A. Ebajemito J. K. Mikkelsen K. B. Bonmati-Carrion M. A. Santhi N. Della Monica C.. (2018). Sleep EEG derived from behind-the-ear electrodes (ceegrid) compared to standard polysomnography: a proof of concept study. Front. Hum. Neurosci. 12:452. 10.3389/fnhum.2018.00452 30534063 PMC6276915

[B60] Swets J. A. Tanner W. P. Jr Birdsall T. G. (1961). Decision processes in perception. Psychol. Rev. 68, 301. 10.1037/h0040547 13774292

[B61] Tomek I. (1976). Two modifications of cnn. IEEE Trans. Syst. Man Cyberne. SMC-6, 769–772. 10.1109/TSMC.1976.430945227295638

[B62] Toyama S. Takano K. Kansaku K. (2012). A non-adhesive solid-gel electrode for a non-invasive brain-machine interface. Front. Neurol. 3:114. 10.3389/fneur.2012.00114 22826701 PMC3399135

[B63] von Lühmann A. Soekadar S. R. Blankertz B. Müller K.-R. (2017). Headgear for mobile neurotechnology: looking into alternatives for EEG and nirs probes, in Proceedings of the 7th Graz Brain-Computer Interface Conference (Graz). p. 496–501. 10.3217/978-3-85125-533-1-92

[B64] Wan Z. Yang R. Huang M. Zeng N. Liu X. (2021). A review on transfer learning in EEG signal analysis. Neurocomputing 421, 1–14. 10.1016/j.neucom.2020.09.017

[B65] Wascher E. Arnau S. Reiser J. E. Rudinger G. Karthaus M. Rinkenauer G.. (2019). Evaluating mental load during realistic driving simulations by means of round the ear electrodes. Front. Neurosci. 13:940. 10.3389/fnins.2019.00940 31551695 PMC6737043

[B66] Whitham E. M. Pope K. J. Fitzgibbon S. P. Lewis T. Clark C. R. Loveless S.. (2007). Scalp electrical recording during paralysis: quantitative evidence that eeg frequencies above 20 hz are contaminated by emg. Clin. Neurophysiol. 118, 1877–1888. 10.1016/j.clinph.2007.04.027 17574912

[B67] Zander T. O. Andreessen L. M. Berg A. Bleuel M. Pawlitzki J. Zawallich L.. (2017). Evaluation of a dry eeg system for application of passive brain-computer interfaces in autonomous driving. Front. Hum. Neurosci. 11:78. 10.3389/fnhum.2017.00078 28293184 PMC5329046

